# Ranolazine Induced Bradycardia, Renal Failure, and Hyperkalemia: A BRASH Syndrome Variant

**DOI:** 10.1155/2019/2740617

**Published:** 2019-12-31

**Authors:** Syed Arsalan Akhter Zaidi, Danial Shaikh, Muhammad Saad, Timothy J. Vittorio

**Affiliations:** ^1^Department of Medicine, BronxCare Health System, New York, NY, USA; ^2^Department of Cardiology, BronxCare Health System, New York, NY, USA

## Abstract

Ranolazine is a well-known antianginal drug, that was first licensed for use in the United States in 2006. It was objectively shown to improve exercise capacity and to lengthen the time to symptom onset in patients with coronary artery disease. The most commonly reported side effects of ranolazine include dizziness, headache, constipation, and nausea. Here, we describe a case of bradycardia, hyperkalemia, and acute renal injury in the setting of ranolazine use. Our patient is an 88-year-old female who presented with abdominal pain, nausea, and vomiting. Her medical comorbidities included hypertension, diabetes, CAD, heart failure with preserved ejection fraction, paroxysmal atrial fibrillation, hypothyroidism, and a history of cerebrovascular accident without any residual deficits. Her prescription regimen included amlodipine, furosemide, isosorbide mononitrate, levothyroxine, metformin, omeprazole, and ranolazine. Physical examination was remarkable for bradycardia and decreased breath sounds in the left lower lung field. Laboratory studies were significant for a serum potassium level of 6.8 mEq/L and a serum creatinine level of 1.6 mg/dL. She was given insulin with dextrose, sodium polystyrene, and calcium gluconate in addition to fluids. Her bradycardia and renal function worsened over the next 24 hours. Ranolazine was discontinued. Metabolic derangements were treated appropriately. After 48 hours from presentation, potassium and renal function returned to baseline and her heart rate improved to a range of 60–100 bpm. She was discharged with an outpatient cardiology follow-up. Ranolazine treatment was not continued upon discharge. In summary, our case illustrates an association between ranolazine and renal failure induced hyperkalemia, leading to conduction delays in the myocardium. Though further studies are warranted, we suspect that this is a variant of the recently described BRASH syndrome. We propose that in cases such as ours, along with treatment of the hyperkalemia, medication review and removal of any offending agent should be considered.

## 1. Introduction

Ranolazine is a well-known antianginal drug, that was first licensed for use in the United States in 2006 [1]. In multiple large randomized control trials, it was objectively shown to improve exercise capacity and to lengthen the time to symptom onset in patients with coronary artery disease (CAD) [1, 2]. It achieves these effects by decreasing myocardial oxygen consumption and causing relaxation, without significantly influencing hemodynamic parameters [2]. The most commonly reported side effects of ranolazine include dizziness, headache, constipation, and nausea (Ranexa PI 2006) [3]. Here, we describe a case of bradycardia, hyperkalemia, and acute renal injury in the setting of ranolazine use.

## 2. Case Presentation

**Figure 1:**
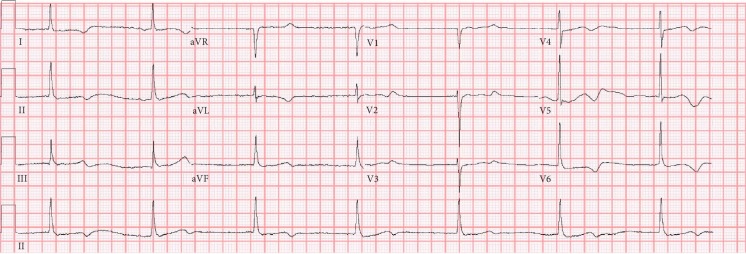
EKG on admission, with a pulse rate of 40 bpm (note the absence of peaked T waves).

An 88-year-old-female presented to our emergency department with complaints of abdominal discomfort associated with nausea and vomiting for one day. Upon arrival, she was afebrile with a blood pressure of 120/54 mmHg, a pulse rate of 40 beats per minute (bpm) (Figure 1), and a respiratory rate of 18 breaths per minute. She denied any chest pain, dyspnea, or palpitations. She denied any recent illnesses, had no history of recent travel, and denied any sick contacts, or recent change in medications. Her medical comorbidities included hypertension (HTN), diabetes, CAD, heart failure with preserved ejection fraction, paroxysmal atrial fibrillation, hypothyroidism, and a history of cerebrovascular accident (CVA) without any residual deficits. Her prescription regimen included amlodipine (10 mg), furosemide (20 mg OD, same dose for past 1 year), isosorbide mononitrate (30 mg OD), levothyroxine (37.5 mcg OD), metformin (500 mg BID), omeprazole (20 mg OD), and ranolazine (500 mg BID, same dose for the past 1 year). Physical examination was remarkable for bradycardia and decreased breath sounds in the left lower lung field, and she was euvolemic on exam. Laboratory studies were significant for a serum potassium level of 6.8 mEq/L and a serum creatinine level of 1.6 mg/dL and eGFR of 32 ml/min/m2 (increased from baseline creatinine of 0.9 mg/dl, eGFR 68 ml/min/m2). Thyroid studies were within the normal range. Chest radiography revealed minimal left pleural effusion. She received an intravenous fluid bolus for her acute renal injury and was administered sodium polystyrene sulfate, calcium gluconate, albuterol, intravenous insulin, and a dextrose injection to treat the hyperkalemia. Over the next few hours, her bradycardia worsened despite correction of the hyperkalemia. A 12-lead electrocardiography (ECG) showed junctional bradycardia at 32 bpm, a normal QRS complex, and a prolonged QTc of 532 milliseconds. There was no demonstrable peaking of the T waves. Atropine was administered without any significant chronotropic response. She was admitted to the cardiac care unit for close monitoring. The following day, her serum creatinine reached a maximum of 2.2 mg/dL. Ranolazine was discontinued. Metabolic derangements were treated appropriately. After 48 hours from presentation, potassium and renal function returned to baseline and her heart rate improved to a range of 60–100 bpm. She was discharged the next day with an outpatient cardiology follow-up. Ranolazine treatment was not continued upon discharge.

## 3. Discussion

Approximately 6.5 million people in the United States alone suffer from chronic angina, with 400,000 new cases identified annually [4]. Almost all such patients are managed with either a beta-adrenergic blocker, calcium channel antagonist, nitrate, or ranolazine, alone or in combination, as indicated by severity of symptoms.

Ranolazine works by inhibiting the slow inactivating component of the cardiac sodium channel during repolarization, and in doing so it reduces the calcium influx via Na^+^-Ca^2+^ exchanger. This reduces myocyte dysfunction in the ischemic heart and improves coronary vascular compliance and resistance [2, 5]. In patients with diabetes mellitus, it has been noted to cause a sustained reduction in glycosylated hemoglobin (hemoglobin A1c) in a dose-dependent manner, with an enhanced effect seen in patients receiving exogenous insulin [6]. Ranolazine is also associated with a dose‐dependent increase in the QT‐interval, with a mean increase of 6 milliseconds at the maximum recommended dosing. Contraindications to ranolazine are prolonged QT‐interval and coadministration with other QT‐prolonging drugs, previous history of ventricular tachycardia, and moderate to severe kidney impairment or severe liver failure [7]. The most common adverse events related to use of ranolazine are headaches (5.5%), dizziness (1% to 6%), constipation (5%), and nausea (≤4%; dose related). Although there is concern over QT prolongation on ECG, its prevalence has been estimated to be less than 1% [8]. Ranolazine can also cause bradycardia, as noted on its FDA package insert. A review of the 5 major ranolazine trials (MARISA [9], CARISA [10], ERICA [11], MERLIN-TIMI 36, and RAN080 [12]) observed that bradycardia is seen in less than 2% of patients.

BRASH syndrome, an acronym for bradycardia, renal failure, atrioventricular (AV) nodal blockage, shock, and hyperkalemia, is a recently coined term for the aforementioned constellation of findings [13]. It has typically been described in patients with underlying cardiac disease on AV nodal blocking agents. Dehydration, gastroenteritis, and poor oral intake [14] have been implicated as the inciting factors, leading to hypotension and prerenal azotemia. The acute renal injury causes hyperkalemia, which in turn hinders AV node activity, when combined with any agent able to induce bradycardia. Bradycardia leads to further kidney hypoperfusion which worsens renal dysfunction and hyperkalemia. This results in a vicious cycle of bradycardia, renal failure, and hyperkalemia. Often clinicians associate hyperkalemia as the sole cause of the bradycardia [15]. The distinction between bradycardia, purely due to hyperkalemia versus that induced by hyperkalemia in synergy with medications, is an important one [16, 17]. In pure hyperkalemic bradycardia, serum potassium levels are higher and ECG changes are common, including broad QRS complexes and peaked T waves [16]. Although in a rare number of cases, such changes might be absent, but usually the absence of such findings in a patient with hyperkalemia and severe junctional bradycardia favors an additional etiology of the bradycardia. The synergism of hyperkalemia with negative dromotropic agents, resulting in bradycardia, is well defined with multiple case reports highlighting such findings in patients on verapamil [18, 19]. We propose that ranolazine can produce a similar effect, thereby leading to a syndrome analogous to BRASH.

A search of the FDA Adverse Event Reporting System (FAERS) database reports 5 patients on ranolazine experiencing bradycardia, renal failure, and hyperkalemia between 2006 and 2018. 4 of the 5 cases required hospitalization and 2 had a “life-threatening” event reported [20]. Although there are reports of syncopal events in patients taking maximal dose or ranolazine at 1000 mg BID and concurrent medications which might lead to increased plasma levels of ranolazine [6], our patient was not on any such concurrent medications nor was observed to have renal dysfunction at baseline. She was also on only 500 mg BID dosing of ranolazine, making it unlikely that the plasma levels of ranolazine were supratherapeutic and lead to any adverse effects due to that.

Our patient was receiving ranolazine for her anginal symptoms and was not on any other negative dromotropic agents, to the best of our knowledge. On review of Google Scholar and PubMed, we could not find any data suggestive of negative dromotropy or negative chronotropy as an adverse effect of ranolazine. Our patient received treatment for hyperkalemia and intravenous fluids for the acute renal injury; however, her symptoms only truly abated once ranolazine was discontinued. At hospital discharge, she was advised to stop ranolazine indefinitely and to continue nitrates for anginal symptoms. We also calculated the Naranjo score for our case—the Naranjo Algorithm, or Adverse Drug Reaction Probability Scale, is a method to assess whether there is a causal relationship between an identified untoward clinical event and a drug using a simple questionnaire to assign probability scores. The Naranjo score for ranolazine in our patient was calculated to be 6 (probable cause) [21].

In summary, our case illustrates an association between ranolazine and renal failure induced hyperkalemia, leading to conduction delays in the myocardium. Though further studies are warranted, we suspect that this is a variant of the recently described BRASH syndrome. We propose that in cases such as ours, along with treatment of the hyperkalemia, medication review and removal of any offending agent should be considered.
